# Commentary: Teleradiology: The Indian Perspective

**DOI:** 10.4103/0971-3026.45338

**Published:** 2009-02

**Authors:** Arjun Kalyanpur

**Affiliations:** Chief Radiologist & CEO of Teleradiology Solution, No: 7G, Council Khata 180/63, Opp. Graphite India, ITPL Main Road, Bangalore, India

The article by Dr.'s Burute and Jankharia[1] brings up some important issues.

In essence, teleradiology in the Indian setting needs to be considered in two completely separate contexts. The first is its ability to provide new economic opportunity for our country. The second and arguably far more significant role lies in its providing a potential solution to the staffing shortages that we, the radiology community in India, face today and will continue to face with increasing severity in the coming years.

In the context of teleradiology as an outsourcing option from more developed countries to India, this is an exciting development in the last decade that has truly put India on the world map of quality healthcare. We have proved to the world, through consistent performance over the past six years, that high quality reporting, of a caliber equal or superior to that provided by western companies, can be delivered from India using the day/night time advantage.[2,3] However, this hard-earned reputation has lately been jeopardized by controversial hard-sell advertising by some newer Indian players. At the Radiology Society of North America (RSNA) meeting that concluded last month, I repeatedly heard from radiologists in the US and Europe who had received mass mailings from providers in India offering ‘low cost, high quality preread’ services. One showed me an example of such an email on his Blackberry, pointing out how badly written the message was, containing numerous typographical and syntax errors. His concern - when an introductory email is itself so shoddy, what quality expectation can there be of the radiology reports? Of even greater concern to him, the email also contained an insinuation that the reports would be “of such high quality that no additional review by a European or American radiologist would be necessary”.

It is imperative to understand that every country has its own licensure requirements. Any preliminary ‘preread’ report would therefore have to be reviewed again by the onsite radiologist. In this context, the article by Burute does not, in my opinion, adequately differentiate between a preread and a so-called ‘ghost’ read. The process of dual review or ‘preread’ in which a preliminary report by a trained professional (radiologist, technologist or physician assistant) is overread by a radiologist licensed in that country, who reviews the study in toto, results in an efficiency, productivity and quality benefit, as has been validated repeatedly in the literature.[4,5] This model is now largely an accepted one - as opposed to the specter that has been raised of “ghost reporting” in which the licensed radiologist does not review the study but simply “signs off”.

Needless to say, such advertisements and offers put our entire country in disrepute and the industry in jeopardy. My recommendation to all who wish to enter the teleradiology providers market is that they should first understand the regulations and implications carefully and thoroughly before they plunge in. International regulations vary greatly from country to country and so what is appropriate in one location may be entirely inappropriate in another. Attending an accredited Teleradiology symposium or session at a national or international conference is one way to familiarize oneself with the issues involved.

The second and to my mind more important discussion relates to the use of teleradiology within India to benefit underserved areas, especially in emergency situations, to increase the reach of subspecialty diagnosis and to ease regional and temporal staffing shortages. Given the grossly inadequate ratio in our country of 1 radiologist to 100,000 people, the use of technology is critical to address these issues. Such efforts may bring great relief and can be done at relatively low cost. A program that we have undertaken with the Ramakrishna Mission hospital in Itanagar, Arunachal Pradesh, has been greatly successful over the past year with subspecialty level reports being available to a poor tribal population.[6] However, at least at this time, a robust business model is not in place for such services within our country, likely due to cost constraints, as Burute and Jankharia note, and so the greatest value is likely to be in the not-for-profit sector (for example, our services to the RKMH are entirely free of charge).

Better utilization of radiologist time and efficiency measures are inherent to the practice of teleradiology, both of which can help to significantly enhance radiologist productivity. No longer is it acceptable for a trained radiologist to expect to report just 10–15 CT scans in a day, something I have observed repeatedly in the recent past years, in the course of conducting applicant interviews. Our goal needs to be to set the bar ever higher both in terms of productivity and quality and the use of technology is an important, if not an essential, part of this endeavor.

To conclude, in my opinion, there are two broad guidelines that we must follow if we are to truly reap the benefits of teleradiology – a) follow the rules and b) let quality and not monetary benefit be our driving force.
